# Tr-Predictior: An Ensemble Transfer Learning Model for Small-Sample Cloud Workload Prediction

**DOI:** 10.3390/e24121770

**Published:** 2022-12-03

**Authors:** Chunhong Liu, Jie Jiao, Weili Li, Jingxiong Wang, Junna Zhang

**Affiliations:** 1 College of Computer and Information Engineering, Henan Normal University, Xinxiang 453007, China; 2 Engineering Lab of Intelligence Business, Xinxiang 453007, China

**Keywords:** cloud data center, transfer entropy, workload forecast, ensemble learning, transfer learning

## Abstract

Accurate workload prediction plays a key role in intelligent scheduling decisions on cloud platforms. There are massive amounts of short-workload sequences in the cloud platform, and the small amount of data and the presence of outliers make accurate workload sequence prediction a challenge. For the above issues, this paper proposes an ensemble learning method based on sample weight transfer and long short-term memory (LSTM), termed as Tr-Predictor. Specifically, a selection method of similar sequences combining time warp edit distance (TWED) and transfer entropy (TE) is proposed to select a source domain dataset with higher similarity for the target workload sequence. Then, we upgrade the basic learner of the ensemble model two-stage TrAdaBoost.R2 to LSTM in the deep model and enhance the ability of the ensemble model to extract sequence features. To optimize the weight adjustment strategy, we adopt a two-stage weight adjustment strategy and select the best weight for the learner according to the sample error and model error. Finally, the above process determines the parameters of the target model and uses the target model to predict the short-task sequences. In the experimental validation, we arbitrarily select nine sets of short-workload data from the Google dataset and three sets of short-workload data from the Alibaba cluster to verify the prediction effectiveness of the proposed algorithm. The experimental results show that compared with the commonly used cloud workload prediction methods Tr-Predictor has higher prediction accuracy on the small-sample workload. The prediction indicators of the ablation experiments show the performance gain of each part in the proposed method.

## 1. Introduction

With the rapid growth of various types of terminal data on mobile platforms, terminal storage capacity is becoming limited. Application deployment to the cloud has become an increasingly common practice in the industry [1]. Elastic and efficient resource management is the characteristic of cloud computing over other computing models [2,3]. Autoscaling and other technologies realize the intelligent scheduling decision of the cloud platform, and the scaling decision of autoscaling is realized based on the prediction result of the workload [4,5]. Over-allocation of computing resources or sudden changes in workload negatively impact resource management. The accuracy of prediction directly affects the efficiency of scaling. The prediction result of workload decides the allocation of various resource requirements, and the accuracy of workload prediction is a key factor affecting cloud resource management [6].

In the cloud platform management system, the monitoring log of the cloud platform will record changes in resource utilization (CPU, memory, I/O, etc.) when each task is running [3,7]. Taking the Google data center as an example, the request of a user is called a job. Each job contains one or more tasks. The monitoring system records at a frequency of sampling every five minutes. There are two types of jobs running in the Google cluster: production priority jobs and batch jobs. Production priorities include longer-duration jobs, and batch jobs are relatively short jobs [8]. This article treats batch jobs that run less than 8 h as short tasks. There are a large number of small-sample workload sequences in the Google data center. Such short tasks also require resource scaling and require fast processing. In regression tasks, short-sequence workload prediction is a small-sample problem. Therefore, the accuracy of short-task workload prediction has an important impact on the performance of autoscaling [9].

The research on time series forecasting provides an effective guarantee for the storage and management of cloud platform resources, and many new and efficient forecasting algorithms are used for resource scheduling [10,11]. Traditional time series forecasting models are fast, efficient and highly interpretable [12]. In this regard, researchers have been improving them and applying them to cloud load forecasting. Daraghmeh et al. [13] applied the Prophet to predict the required resource utilization of workloads. Shi et al. [14] used BHT_ARIMA to predict the short-term workload. The prediction models based on machine learning are widely used in cloud workload prediction because of their strong learning and prediction abilities for burst data [15,16]. Liu et al. [17] proposed an adaptive prediction method. Ouhame et al. [18] proposed CNN-LSTM to model irregular information in short sequences. Based on the above research status of cloud workload forecasting, traditional time series forecasting algorithms are suitable for sequences with periodic and obvious trends. Machine-learning-based workload prediction algorithms require a large amount of training data. However, they have no obvious advantage in the forecasting effect of small-sample workload sequences with strong burstiness. At present, there are few systematic studies on the problem of small-sample workload sequence forecasting.

Small sample workload forecasting has the following challenges:

(1) The sample points of the workload sequence targeted are too short (less than 100 sample points) compared to other prediction work, and the corresponding number of tasks is small (mostly less than 200). The available sample size is small. Such workload prediction can be viewed as a small-scale sample problem. For such problems, although some solutions have been proposed, most of them are coarse-grained and cannot fully excavate and effectively utilize the data information in the cloud platform.
(2) Small sample sequences have strong irregular mutation, and the data lengths are quite different in domains, which leads to increased difficulty in the representation of sequence workload features. Task sequences often have different patterns of change. There is no apparent periodicity (about 90% of tasks are aperiodic).

Compared with traditional forecasting and machine learning forecasting methods, the forecasting model based on transfer learning has received more and more attention due to its excellent effect. The initial performance of the transfer learning model is higher, and the convergence is better. Antoine et al. [19] proposed a domain adaptation method for regression tasks, called WANN. Xu et al. [20] proposed a two-stage transfer prediction algorithm for short-term charges, which combined the time series trend decomposition with the two-stage TrAdaBoost.R2 to improve the prediction accuracy.

In recent years, the research value of small-sample problems in practical problems has been constantly explored [21]. Scholars in related fields put forward new work that is not advanced [22,23]. The research in [24,25] shows that transfer learning is an effective method to solve the problem of small samples, and there is a certain similarity in the changing trend between medium tasks and short tasks. In transfer learning training, the training process is robust, and the generalization ability of the model is stronger. Therefore, this paper uses transfer learning to predict small-sample workload sequences and obtain better accurate prediction results.

To address the above challenges, we propose a sample weight transfer ensemble algorithm for small-sample workload sequences, denoted as Tr-Predictor. In workload prediction work, the fusion of ordinary linear models with migration algorithms often fails to obtain better prediction results on small-sample workload sequences. For the irregularity and mutability of short-task workload sequences, the deep learning algorithm is incorporated into the sample weight migration algorithm to obtain better prediction accuracy by capturing the potential features of the sequences. The main contributions of this paper are as follows:

- A two-stage transfer ensemble prediction algorithm is proposed to predict the small-sample workload sequences with little historical data, weak trend, and periodicity. It integrates the advantage of the deep network and shallow model. The weight transfer method obtains relatively more long-term trend features from the source domain to assist the prediction of target data.
- A fusion time series similarity measurement criterion is proposed to measure the similarity between the source domain and the target domain. The measurement method uses TWED and TE to measure the correlation between domains and selects the suitable medium length set that serves as the source domain. There is a certain causal relationship between the selected source domain and the target domain. A suitable source domain can effectively improve the prediction accuracy of transfer learning.
- The algorithm is verified on the monitoring logs of two public large-scale general cloud data centers. A total of 12 groups of small-sample workload data with different complex change patterns is selected and compared with the current state-of-the-art workload prediction algorithms. The extraction algorithm has the characteristics of high precision and universality on small-sample data.

The rest of this paper is organized as follows. In Section 2, we briefly introduce the related techniques involved in the proposed method. In Section 3, the proposed method is presented in detail. Section 4 conducts experiments and corresponding analyses. Finally, our work is summarized in Section 5.

## 2. Preliminary

This section mainly introduces three parts of related technologies: time warp edit distance (TWED), AdaBoost-LSTM and transfer entropy (TE). The techniques in Section 2.1 and Section 2.3 are used for the selection of similar workloads, and the middle section is used as the theoretical basis for the prediction method proposed in this paper.

### 2.1. TWED

The most commonly used time series similarity measures include Euclidean distance and dynamic time warping. Euclidean distance is fast and efficient, but it is susceptible to time shifts. Dynamic time warping is the most widely used and improved algorithm, but it cannot handle time series with different sampling rates. Dynamic time warping will not perform as well for source and target domain data with different sampling rates. Therefore, we use TWED, which incorporates a ‘stiffness’ [26] parameter as an indicator to control the elasticity of the metric [27], allowing more flexibility in matching between sequences. As TWED takes into account differences in timestamps, it can better solve the problem of time shifts in the sequence matching process and can be used on time series data with different sampling rates. The algorithm can efficiently retrieve time series while adjusting the elastic measurement parameters [28].

DTW is used to calculate the similarity between two time series, which is characterized by allowing time scaling during matching. In voice, picture, signal, medical and other fields, DTW has shown ideal results. However, we ultimately choose TWED as the similarity independent criterion between workload sequences. The reasons are as follows:

- TWED takes into account the difference of timestamps so that it can be used for workload sequence data with different sampling rates. For cloud platform data with complex data, TWED is more robust than DTW in the workload sequence measurement;
- Compared with DTW, TWED can use trigonometric inequality to speed up the search in metric space;
- Based on strict evaluation experiment research in [26,27], it is shown that TWED shows higher classification accuracy than DTW in the classification of multiple groups of open time series datasets, which further reflects the advantages of TWED.

Suppose a time series of length *m*: $P_{1}^{m}=\left\{\left(p_{1},t_{p1}\right),\ldots \left(p_{i},t_{pi}\right),\ldots \left(p_{m},t_{pm}\right)\right\}$ and another time series of length *n*: $Q_{1}^{n}=\left\{\left(q_{1},t_{q1}\right),\ldots \left(q_{j},t_{qj}\right),\ldots \left(q_{n},t_{qn}\right)\right\}$. The calculation formula of TWED is as follows:

(1) $$\begin{array}{c} d_{twed}\left(P_{1}^{m},Q_{1}^{n}\right)\\ =\min\left\{\begin{array}{c} d_{twed}\left(P_{1}^{m-1},Q_{1}^{n}\right)+d_{LP}\left(p_{m}-p_{m-1}\right)\\ +v\cdot (t_{pm}-t_{pm-1})+\lambda \\ d_{twed}\left(P_{1}^{m-1},Q_{1}^{n-1}\right)+d_{LP}\left(p_{m},q_{n}\right)\\ +d{}_{LP}\left(p_{m-1}-q_{n-1}\right)\\ +v\cdot (|t_{pm}-t_{qn}|+|t_{pm-1}-t_{qn-1}|)\\ d_{twed}\left(P,Q_{1}^{n-1}\right)+d_{LP}\left(q_{n}-q_{n-1}\right)+\\ v\cdot (t_{qn}-t_{qn-1})+\lambda \end{array}\right. \end{array}$$

where $t_{qj}$ and $t_{pi}$ represent the timestamps corresponding to the two time series sequences, $d_{LP}$ denotes LP-norm, and λ and *v* are two nonnegative parameters used to adjust the metric “stiffness”.

### 2.2. AdaBoost-LSTM

Adaptive boosting (AdaBoost) is a typical regression algorithm that linearly adds a series of weak estimators to obtain strong estimators through the idea of ensemble. The sample weight transfer algorithm TrAdaBoost.R2 is an extended algorithm based on the AdaBoost.R2 [29], supplementing the data of the source domain to help build the model. It improves the accuracy of selecting the next weak learner at each iteration by adjusting the weights of the training samples. We use the AdaBoost to integrate a set of LSTM [30] predictors to improve the fitting ability of the regression model [31]. The AdaBoost-LSTM model is shown in Algorithm 1.

### 2.3. Transfer Entropy

There is not only a correlation among time series variables, but also a certain causal relationship. The essence of TE [32] is conditional mutual information. It is an indicator to measure the directional transfer of information between two time series [33]. Certain causal relationships can improve the impact on prediction results. Utilizing a nonparametric method for estimating TE via Copula Entropy(CE) requires only two simple steps and is more computationally efficient [34]. The transfer of information among workloads is studied, and the asymmetry is detected to assist in constructing the corresponding driving and response relationships. The TE between the series *P* and *Q* is:

(2) $$TE=\sum p\left(Q_{i+1},Q^{i},P_{i}\right)\log\frac{p\left(Q_{i+1}|Q^{i},P_{i}\right)}{p\left(Q_{i+1}|Q^{i}\right)}$$

where $Q^{i}=\left(Q_{1},\ldots ,Q_{i}\right)$;

When TE is only represented by CE:

(3) $$\begin{array}{c} TE=-H_{c}\left(Q_{i+1},Q^{i},P_{i}\right)+H_{c}\left(Q_{i+1},Q^{i}\right)\\ +H_{c}\left(Q^{i},P_{i}\right)-H_{c}\left(Q^{i}\right) \end{array}$$

**Algorithm 1** AdaBoost-LSTM**Input:** The labeled target time series sequence, *T*, of size *n*$T=(x_{1},x_{2},\ldots ,x_{n})$, the maximum number of iterations, *N* and a base learning algorithm, Learner LSTM. Set the initial weight vector: Wjt=11nn.**Output:** Strong learner $y_{f}\left(x\right)$ is equal to the $LSTM_{t}$ prediction result $y_{t}\left(x\right)$ and its corresponding $\beta_{t}$ generated by weight collection. 1:Call learner $LSTM_{t}$ with the training set, *T*, according to the distribution, $W_{j}^{t}$, to train and give the hypothesis, $y_{t}:x\to R$. 2:Calculate the adjusted error for every sample:
$$D_{t}=\max_{j=1}^{n}\left|y_{j}-LSTM_{k}\left(x_{j}^{}\right)\right|$$
$$e_{j}^{t}=\frac{{\left(y_{j}-LSTM_{N}\left(x_{j}\right)\right)}^{2}}{{\left(D_{t}\right)}^{2}}$$ 3:Calculate the adjusted error of the $LSTM_{t}$ model:
$$\varepsilon_{t}=\sum_{j=1}^{n}e_{j}^{t}w_{j}^{t}$$if $\varepsilon_{t}\geq 0.5$, stop and set N=t−1. 4:Let $\beta_{t}=\varepsilon_{t}/\left(1-\varepsilon_{t}\right)$. Update the weight vector: $w_{j}^{t+1}=w_{j}^{t}\beta_{t}^{1-e_{j}^{t}}/Z_{t}$.($Z_{t}$ is a normalizing constant.) 5:Loop step 1 to 4. Reserve all models: $LSTM_{1},\ldots LSTM_{t},\ldots LSTM_{N}$.

## 3. Methodology

**Figure 1:**
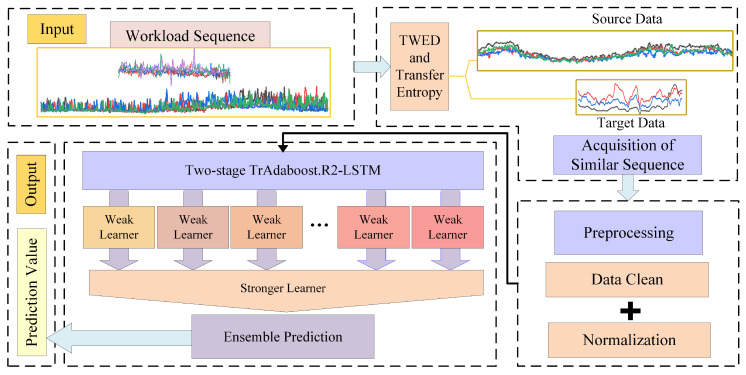
Flowchart of the proposed approach Tr-predictior.

Tr-predictior consists of the following parts: extracting the required workload from the dataset, using TWED and TE to obtain a set of similar sequences in the source and target domains, preprocessing the resulting data for cleaning and normalization, using the prediction model to obtain the final predicted value of the small-sample workloads and finally outputting the prediction value. The specific flow of the proposed method is shown in Figure 1.

### 3.1. Acquisition of Similar Sequences

In our method, the combination of TEWD and TE is used to select the appropriate source domain for the following reasons. TEWD allows time-shifted elasticity measures. When considering time series information retrieval, objects in metric space can be efficiently indexed and retrieved, and local time-shifting is supported. The TWED proposes a lower bound that allows linking the matching evaluations of two time series to the downsampled representation space and linking their matching evaluations to their original representation space. TE is employed to further evaluate the distributional similarity between domains. As a causality measurement tool beyond association, it can measure not only the similarity between two probability distributions but also the degree of influence of the source domain on the target task. Our method of obtaining similar sequences is a measure based on similarity and causality.

Compared with feature-based similarity measures, our quantification standard will be faster and more convenient. TWED is more robust than other methods due to its consideration of the timestamp. The measurement method we propose considers the similarity between time series and the amount of information transferred, selecting an appropriate source set for the target task. Using the TE to judge the causal relationship and measure the similarity between domains is more conducive to improve the prediction accuracy of transfer learning.

The inherent information with similar distributions in the source and target domains is more valuable than other data. Based on the special data type targeted in this paper, there is inconsistency in the data distribution of the source and target domains. In addition, the assumption that the data is independent and identically distributed is not satisfied; therefore, it is difficult for traditional models to adapt to the changes in data distribution dynamically. By improving the inconsistency of data distribution, the effect of transfer prediction can be further improved.

### 3.2. Data Preprocess

The source and target domain data are cleaned and extracted from the cloud dataset. For missing values in the data, the last observation before the missing value is used to fill in. We use the 3σ-rule to identify and remove outliers in the dataset. As for sequence $T=\{t_{1},t_{2},\ldots ,t_{n}\}$, we calculate its residual error $v_{i}=t_{i}-t\left(i=1,2,\ldots ,n\right)$; *t* refers to the arithmetic mean, and the standard error σ is calculated according to the Bessel formula. If the residual error $v_{b}\left(1\leq b\leq n\right)$ of $t_{b}$ conforms to Formula (4)   

(4) $$|v_{b}\left|\;=\;\right|t_{b}-t|>3\sigma ,$$

then $x_{b}$ is considered to be an outlier and eliminated. To eliminate the difference among the data dimensions, the sequence is normalized. The normalized sequence is $T^{*}$. $T_{x}$ and $T_{n}$ represent the maximum and minimum values in *T* as shown in Formulas (5) and (6):

(5) $$T_{std}=\frac{T-T_{n}}{T_{x}-T_{n}}$$

(6) $$T^{*}=T_{std}*\left(T_{x}-T_{n}\right)+T_{n}$$

### 3.3. Sample Weight Transfer Ensemble Approach

The proposed method is based on Algorithm 1 and two-stage TrAdaBoost.R2 [35] using the ensemble idea of AdaBoost in transfer learning. This facilitates increasing the weight of target domain instances with low error rates, so strong regressors have a more important decisive role in ensemble learning.

Since the single learner LSTM has a weak predictive ability for small-sample workloads in theory, the integrated operation makes the optimal effect learned by every single learner superimposed. Then, the final model can be trained as a strong learner. LSTM captures temporal dependency by learning deep features at different levels in each iteration. Two-stage TrAdaBoost.R2 mixes the sample data from the source and target domains to construct a training set as the input of the model. After computing the learning error of the previous weak learner in the test set correlated between the source and target domains, the new learning error is used to reupdate the sample weights in the current weak learner. The proposed algorithm can be described as Algorithm 2.
**Algorithm 2** Tr-Predictior**Input:** Suppose $T=\{t_{1},t_{2},\ldots ,t_{n}\}$ is a set containing multiple workload sequences of different lengths and the base learning learner LSTM.**Output:** The ensemble transfer model obtains the predicted value through the following formula:$Temp_{j}=\frac{\log\left(\frac{1}{\beta_{t}^{j}}\right)}{\sum_{j=1}^{N}\log\left(\frac{1}{\beta_{t}^{j}}\right)LSTM_{j}\left(x\right)}$**For** t = 1,2,…,S. 1:Call Equations (1) and (3) to obtain the source domain data, recorded as $T_{source}$ (length n); 2:Call Equations (4)–(6) to preprocess data, extract target data as $T_{target}$ (length m), $T_{c}=T_{source}+T_{target}$. 3:Set the initial weight vector:Wt=11m+nm+n,i=1,2,…,m+n. 4:Set the total weight of $T_{source}$: $w_{i}^{s}=n/\left(m+n\right)$. The total weight of $T_{target}$ is $w_{i}^{t}=m/\left(m+n\right)T_{source}$. 5:Call the AdaBoost-LSTM (Algorithm 1) to train data $T_{c}$, freeze the weight $w_{i}^{s}$ of the top *n* source data in the process of training and only update the weight of the target $w_{i}^{t}$, record the above training model as $model_{t}$. (Train hypothesis in $model_{t}$ for $LSTM_{t}$ is $h_{t}:x\to R$.) 6:Use Step 2 in Algorithm 1 to calculate the adjusted error of each instance in the target domain: $e_{i}^{t}$, change $\beta_{t}$ in algorithm 1 to $\beta_{s}=1/\left(1+\sqrt{2\log\left(\frac{n}{N}\right)}\right)$, use it to calculate the adjusted error of each instance in the source domain: $e_{i}^{s}$. 7:Calculate the adjusted error, $\varepsilon_{t}$, of $model_{t}$:$\varepsilon_{t}=\sum_{i=1}^{n}e_{i}^{t}w_{i}^{t}$; if $\varepsilon_{t}\geq 0.5$, stop and set N=t−1. 8:Let $\beta_{t}=\varepsilon_{t}/\left(1-\varepsilon_{t}\right)$, freeze the weight of the target domain, then just update the weight vector of the new source domain: $w_{i}^{s+1}=w_{i}^{s}\beta_{t}^{e_{i}^{t}}/Z_{t}$.($Z_{t}$ is a normalizing constant.)**End for**. 9:**return**$y^{pre}=\sum_{j=1}^{N}Temp_{j}$.

The algorithm proposed in this paper has two advantages.

- First, it replaces shallow weak learners with deep learning algorithms. Two-stage TrAdaBoost.R2 extracts the weight distribution information of similar data from the source domain and builds a stronger model than AdaBoost. The default weak learner in two-stage TrAdaBoost.R2 is linear regression. However, the linear regression model needs cross-validation to determine the optimal model after learning the mapping function. The deep learning algorithm can learn the optimal weight through the constraints formed by its loss function, and dropout can solve overfitting problems in the neural network. We combine LSTM with two-stage TrAdaBoost.R2. The new basic regressor can learn the characteristics of long-term series data in the source domain and update the sample weights preferably compared with the linear model.
- Second is the two-stage weight adjustment strategy. Two-stage TrAdaBoost.R2 can solve the extreme weight distribution of training data when TrAdaBoost updates the weight and effectively avoid the overfitting problem of the model. The algorithm is adjusted to a two-stage strategy for updating the weights at the time of execution. The main content of the first stage is reflected in Step 5 of Algorithm 2, which is further described here. Set the initial weight of the source domain and target domain instances, integrate the source domain and target domain to obtain $T_{c}$ and apply AdaBoost-LSTM to train $T_{c}$. The total weight is 1, and *n* is far greater than *m*. Multiple iterations will cause the target domain weight to be close to the source domain instance weight and tilt. To avoid this problem, the weights of the first *n* instances ($T_{source}$) are frozen in the training process, and only the weights of $T_{target}$ are updated. The main content of the second stage is reflected in Step 8 of Algorithm 2: the weights of the source instances are continuously adjusted downward until the LSTM reaches some optimal point in the iterative process. In addition, this model keeps the weights of the target instances unchanged. Then, the model only reserves the assumptions generated in the second stage to determine the output integration result. The most similar target data with source instances are utilized in this way, ignoring source instances that differ from target data.

## 4. Experiments

In this section, we introduce the datasets used in the experiment, the comparison algorithms and the evaluation indicators. Experiments are divided into three groups: visualization of similar workloads, prediction results of small-sample workload sequences and finally the ablation experiments on different components in the proposed method.

### 4.1. Experiment Preparation

This study is aimed at the small-sample workload in the Google Cloud dataset, but to verify the performance of the multiple mention algorithm, another comparison is made on the public Alibaba cluster-trace.

The public dataset Google trace data [36] records the logging of the Google Cloud Platform for 29 days in 2011, including about 672,074 jobs and 26 million tasks [37]. Data such as CPU and disk utilization are sampled every five minutes. This paper selects the mean CPU usage rate data under the task resource usage table for experiments and takes the data of the average CPU utilization under nine different jobs for experiments. The data used in the experiment are shown in Table 1.

The comparative dataset is verified with real workload trace data from Alibaba cluster-trace [38], which includes data records of 4000 machines, and the runtime resource usage time is eight days. To improve the utilization rate of overall resources, Alibaba has continuously opened up the real data of the cloud platform for scholars to study. In the 2018 Alibaba Open Cluster, the batch workload on each computer is included. In the experiments, CPU usage was used as the main performance indicator of the workload. Three sets of small-sample sequences were extracted and recorded as machine_A, machine_B and machine_C; their corresponding sampling lengths were 100, 85 and 96, respectively. The prediction object we compared was the CPU utilization recorded on each machine, and its record range was [0–100].

The sliding window was used to construct training samples and test samples. In this experiment, the sliding window size was set among 3–6, the dropout was 0.2, and the learning rate was 0.001. In the experiment, the comparison algorithm adopted the traditional cloud workload prediction model ARIMA [39], small-sample prediction model BHT_ARIMA [40], CNN-LSTM-Attention (CLA) and CNN_LSTM [18] and weighted countermeasure networks WANN [13] and AdaBoost.R2 [41].

### 4.2. Evaluation Indicators

In our experiment, four commonly used evaluation indicators were used to evaluate the cloud platform workload prediction method proposed in this paper and other comparison algorithms, including Mean Absolute Percentage Error (MAPE) [42], Mean Absolute Error (MAE) [43], Mean Squared Error (MSE) [44] and R Squared ($R^{2}$) [45]. The smaller the value of the first three metrics, the better model fit. The value of $R^{2}$ was closer to one, which proves that the model performance is better. The true value of the sequence was recorded as $y_{i}$, and the predicted value was denoted as ${\hat{y}}_{i}$. Here, ${\bar{y}}_{i}$ is the mean value corresponding to the sampling points in the sequence from one to n, and i={1,2,…,n} represents the i-th sample. The four indicators were calculated as follows:

(7) $$\mathrm{MAPE}\left(y,\hat{y}\right)=\frac{1}{n}\sum_{i=1}^{n}\frac{\left|\right|y_{i}-{\hat{y}}_{i}\left|\right|}{\left|\right|y_{i}\left|\right|},$$

(8) $$\mathrm{MAE}\left(y,\hat{y}\right)=\frac{1}{n}\sum_{i=1}^{n}|y_{i}-{\hat{y}}_{i}|,$$

(9) $$\mathrm{MSE}\left(y,\hat{y}\right)=\frac{1}{n}\sum_{i=1}^{n}{\left(y_{i}-{\hat{y}}_{i}\right)}^{2},$$

(10) $$R^{2}\left(y,\hat{y}\right)=1-\frac{{\sum {}_{i=1}^{n}\left(y_{i}-{\hat{y}}_{i}\right)}^{2}}{{\sum {}_{i=0}^{n}\left(y_{i}-{\bar{y}}_{i}\right)}^{2}}.$$

MAPE represents the deviation of the prediction results from the actual value. MAE reflects the actual situation of the predicted value error, MSE measures the difference between the actual value and the predictive value, and $R^{2}$ reveals the gap between the prediction value and the ideal situation.

### 4.3. Experimental Results and Analysis

#### 4.3.1. Extraction of Similar Sequences

Taking the workload data under Job H extracted from the Google dataset as an example, we drew the probability distribution diagrams of similar workloads before and after the acquisition, respectively.

**Figure 2:**
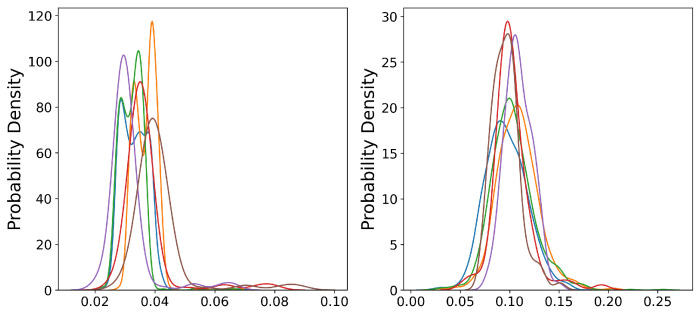
Probability distribution plots for random and similar sequences. The different coloured curves in the two subplots indicate the probability distributions for different workload utilisation.

It can be seen from Figure 2 that although different sequences come from the same dataset, their probability density distributions have obvious differences, which violates the theoretical premise of independent and identically distributed conditions of most machine learning algorithms and may cause negative transfer results. However, after the similarity measurement, the probability density distribution between similar workloads tends to be consistent, which eliminates the problem of data distribution inconsistency among different workloads, thereby improving the prediction effect of transfer learning.

**Figure 3:**
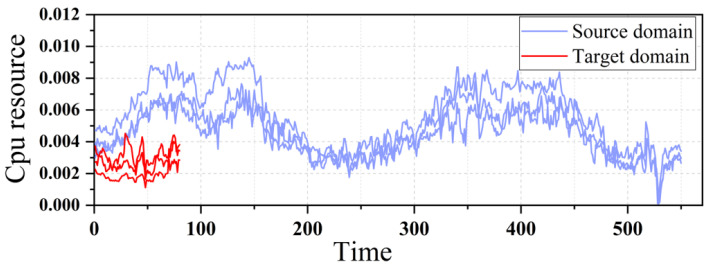
Similar sequences of source domain and target domain.

Figure 3 is a visual trend diagram of similar data sequences in the source and target domains. The blue lines in the figure represent long task sequences in the source domain, and the red lines are short-task sequences in the target domain. We select the four most similar sequences in the source and target domains. Although the data of the source and the target domain have more differences in the length of sampling points, after measuring them through the similarity algorithm, different sequences with high overall similarity and a certain causal relationship can be obtained.

#### 4.3.2. Predictive Result Analysis of Tasks

(a) Alibaba dataset.

Three groups of small-sample data extracted from the Alibaba dataset were subjected to comparative experiments. There are six kinds of comparison algorithms. The comparison of evaluation indicators and predicted values was carried out to verify the universality of the algorithm proposed in this paper on small-sample workload data.

By analyzing Table 2, Table 3 and Table 4 on the three sets of data in the Alibaba dataset, Tr-Predictor performs optimally under the MSE and MAPE indicators, indicating that our prediction results are relatively accurate. Under the MAE indicator, the ARIMA algorithm performed better on the Machine_B dataset, which shows that ARIMA has certain competitiveness in the appropriate data type. The MAPE value of the WANN in these tables is the second best. Due to the strong periodicity of the data for this task, ARIMA is more suitable for its data characteristics. Furthermore, we note that WANN also performs well under the MAPE metric, which indicates that the transfer learning framework performs relatively well.

**Figure 4:**
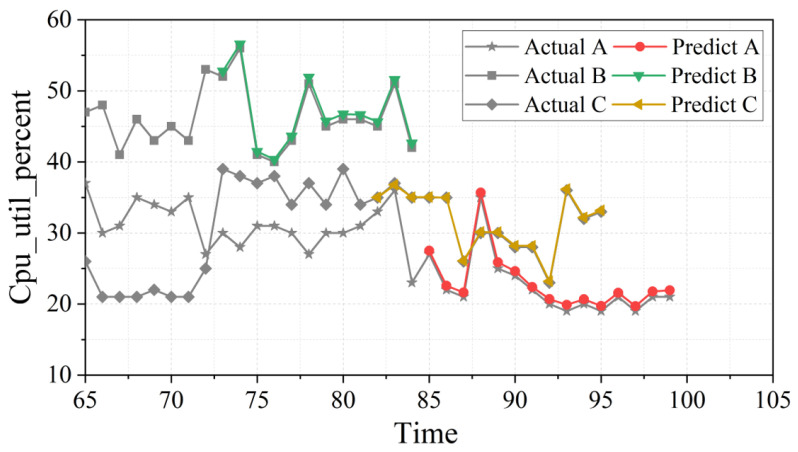
Prediction results of 3 groups of small-sample workload sequences under the Tr-Predictor.

As we can see from Figure 4, the Tr-Predictor algorithm proposed in this study can still have a good prediction effect for the complex change pattern among multiple workloads in the Alibaba cluster-trace. The fitting effect of small-sample workload sequences of different lengths is excellent, which is almost close to the real value of the workload. Compared with the Google dataset, the complex data of the Alibaba cloud is more stable, and the method proposed in this paper can still fit well at the peak point.

(b) Google dataset.

**Figure 5:**
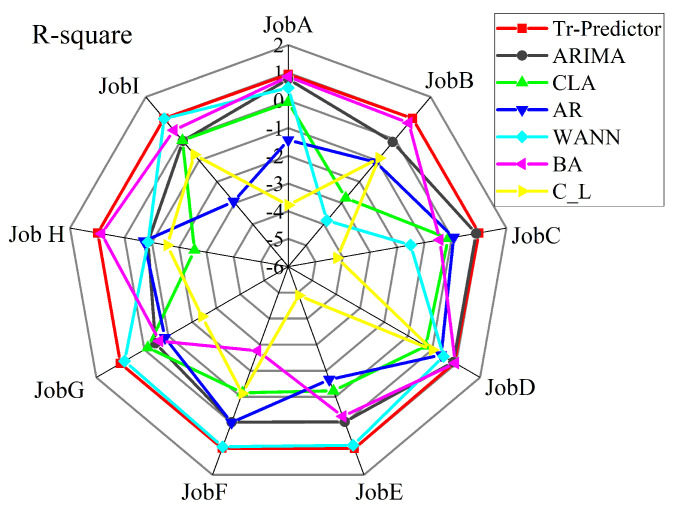
$R^{2}$ evaluation index of comparing algorithms.

In this group of experiments, the task data used are from nine different jobs in Table 1, which contains different kinds of workloads with obvious aperiodicity and strong mutation. To verify the excellent performance of the proposed algorithm on small-sample cloud workload data, nine groups of complex and diverse cloud data were selected for experiments. The results of each algorithm under the MAE, MSE and MAPE indicators are shown in Table 5, Table 6 and Table 7, respectively. Figure 5 shows the results of each algorithm under the $R^{2}$ indicator, and the prediction results are further analyzed.

As shown in Table 5, the proposed method outperforms other baseline models on the MAE metric. The performance of ARIMA on these nine sets of workload data was uneven, as it was heavily influenced by the periodic nature of the data. Due to the particularity of the small-sample workload, the insufficient amount of training data leads to poor performance of deep learning algorithms, such as AdaBoost.R2 and CNN_LSTM. The BA algorithm proposed by Huawei Lab for small-sample workload prediction outperforms other baseline algorithms. The method Tr-Predictor proposed in this paper has a higher accuracy rate than the tensor ARIMA algorithm on these task datasets. It is because the integrated strategy makes the results stronger and the prediction effect better. The performance of the Tr-Predictor is better than all baselines, and the joint training using source domain data and target domain is more accurate than non-migration prediction.

From the result of Table 6, it is not difficult to see that the distribution of the results of these nine sets of data under the MSE evaluation index is similar to that of Table 5. Since the data training samples of the target domain used in this experiment are few, and only the CNN_LSTM model is used without transfer learning, the prediction accuracy will be greatly affected. Therefore, there is a big gap between the prediction effect of the CNN_LSTM network and the ensemble algorithm proposed in this paper. Compared with the BA algorithm, the MSE index of the Tr-Predictor algorithm in this table has a greater reduction rate than the MAE index. The source data is favorable for predicting the target data.

Based on the MAPE indicators in Table 7 and the above MAE and MSE indicators, the Tr-Predictor algorithm can achieve the minimum value under these indicators. This algorithm can open the gap with other algorithms in different types of task data. The prediction effect of the BA algorithm is better than the general algorithm. However, due to the double limitation of the number of sampling points and the number of load sequence training, the BA algorithm still has some gaps from the algorithm proposed in this paper. The experimental evaluation scores verify the advantages of transfer learning and integrated hybrid algorithms. The prediction performance of the hybrid ensemble learning method is better than a single model. Ensemble learning can significantly improve the prediction performance of a single model. Therefore, the Tr-Predictor algorithm will be able to use medium and long series to assist in predicting short series and realize the effective transfer of trend information between workloads.

The optimal score of the regression model in the $R^{2}$ evaluation function is one, but the effect of the model will be arbitrarily degraded by the influence of the training dataset, and the value of $R^{2}$ may also be negative. Under the $R^{2}$ evaluation index, the scores of each prediction algorithm in the experiment are shown in the form of radar charts as shown in Figure 5. The results obtained by the three algorithms of AdaBoost.R2, CNN_LSTM and CLA on this index are all negative values. The scores of BHT_ARIMA, ARIMA and Tr-Predictor are among zero and one. The red line in the outermost circle in the radar chart is almost close to one, indicating that the $R^{2}$ index value of the Tr-Predictor algorithm is closest to one, which proves that the prediction performance of the model is better.

**Figure 6:**
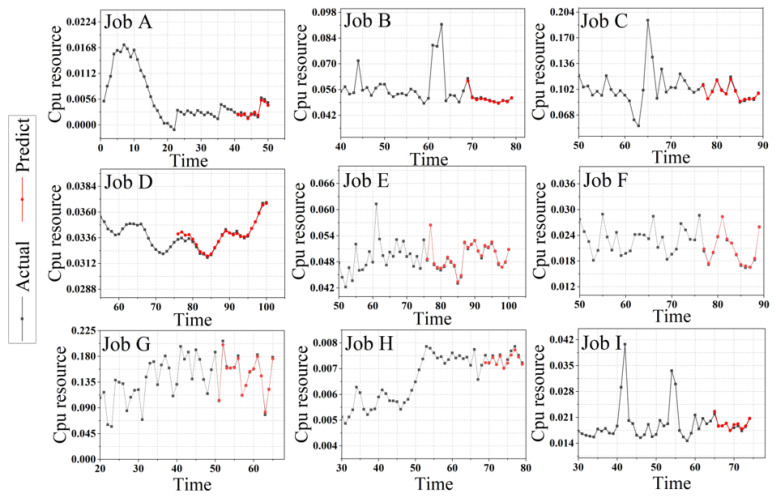
Prediction results of 9 groups of small-sample workload sequences under the Tr-Predictor.

The comparison experiments for the above nine groups of tasks are recorded as follows. Figure 6 is the prediction effect diagram of the nine groups of data in which the black line represents the original data, and the red line represents the predicted value of the target domain.

It can be seen from the forecast trend graph of each job that the general trend of the predicted value of the target domain is consistent with the trend of the original value, but for the peaks in each part of the time series graph, especially the highest point, there is still a difference between the predicted value and the actual value. There is a certain gap. For the points among the peaks, the Tr-Predictor algorithm can accurately predict, and the fitting degree is relatively high.

According to the prediction trend chart of different task data, it can be concluded that compared with the irregular task data, the task data with obvious periodicity can predict the peak more accurately. There is still space for improvement in the prediction of the highest and lowest points.

The experimental results corresponding to different task data in the above figures display the change in data characteristics that will make the prediction results of the same algorithm different. Synthesizing the prediction results shown in Figure 6, the performance of the algorithm for cloud platform small-sample workload data is relatively excellent. We observed in the figure that with the mutation of the workload sample point, the Tr-Predictor prediction effect on the peak point is not significant.

#### 4.3.3. Ablation Experiment

This part shows the performance gain of each part of the Tr-Predictor algorithm module through the following comparative experiments. The set of data from Google center-trace data was selected for comparison randomly. Here, the four regression indicators in Section 4.2 were still used as evaluation criteria for ablation experiments. In the experiment, we verified the improvement of different components in the algorithm from three aspects: the improvement of weak learning, the two-stage weight update strategy and the effect of transfer learning.

As shown in this ablation experiment, w/o means that the entity nodes proposed in this chapter are not used in this model, w/o LSTM means that the weak learner in the ensemble algorithm is the default linear regression, w/o Tr means that only the ensemble is used. The learning algorithm AdaBoost-LSTM makes predictions without adding transfer learning; w/o TB means no ensemble strategy.

It can be seen from the ablation experiment results in Table 8, Table 9 and Table 10 that effective positive transfer can greatly improve the accuracy of workload prediction, and for small-sample workload data, the replacement of weak learners and the deep network LSTM can better capture the dependency among workloads. Shallow models struggle to capture complex patterns of variation among workloads. The deep network can learn different characteristics in multiple iterations to achieve an effective prediction. The combination of different components in the proposed algorithm can achieve better prediction accuracy.

## 5. Conclusions

The accuracy of cloud platform resource demand prediction has important economic benefits and application value. It is significant for improving the utilization of cloud computing equipment center resources and alleviating the storage pressure of mobile terminals. This paper studies LSTM as a nested base learner in ensemble learning. We integrate it with the two-stage TrAdaBoost.R2, which takes full advantage of transfer learning and the LSTM model. Among them, LSTM is used as a regression tool, and the two-stage TrAdaBoost.R2 algorithm is used for model enhancement. Based on the above viewpoints, this paper proposes the cloud platform workload prediction integration algorithm Tr-Predictor based on sample weight transfer, which is used for the prediction of small-sample workload sequences. In this algorithm, TWED and TE are used to find source domain datasets similar to the target domain. The ensemble algorithm is used to effectively transfer the dependency and characteristic information of the workload sequence. Important and different time series features are learned through each iteration. The proposed method combines the prediction results of the weak learner with the weights to obtain the final effect. Different from other methods, this method has the characteristics of universality and high precision for small-sample data in the cloud platform. Effective prediction of small-sample workload sequences is of great significance to resource management and scheduling, and the algorithm has essential practical application value for elastic resource management in cloud platforms.

In the future, improvement in the complexity and running time of the ensemble algorithm will be considered. We will try some learnable distance parameterized by neural networks, set the learned metric to replace TWED and try to use other more suitable deep learning algorithms as base learners.

## Figures and Tables

**Table 1:** Experimental data.

Job ID	Number of Tasks	Length Range Value
Job A: 17109330	172	[50,312]
Job B: 5544435560	512	[80,1000]
Job C: 6239009799	90	[90,997]
Job D: 6280685099	233	[50,313]
Job E: 4969889774	100	[100,500]
Job F: 5905891756	10	[80,550]
Job G: 3996806186	71	[65,552]
Job H: 5063960317	180	[50,80]
Job I: 6176114691	290	[75,551]

**Table 2:** Prediction Results of machine_A.

Algorithm	MAE	MSE	MAPE	$R^{2}$
Tr-Predictor	$1.42\times 10^{-1}$	$3.01\times 10^{-2}$	$9.01\times 10^{-3}$	$9.967\times 10^{-1}$
ARIMA	3.205	17.87	$3.42\times 10^{-1}$	$3.815\times 10^{-1}$
CLA	$2.09\times 10^{-1}$	$5.301\times 10^{-2}$	5.593	−1.056
AR	3.847	21.35	$1.68\times 10^{-1}$	$2.74\times 10^{-1}$
WANN	2.966	11.77	$1.25\times 10^{-1}$	$5.92\times 10^{-1}$
BA	$2.96\times 10^{-1}$	$2.22\times 10^{-1}$	$2.49\times 10^{-1}$	$9.99\times 10^{-1}$
C_L	$2.62\times 10^{-1}$	$9.30\times 10^{-2}$	$7.86\times 10^{-1}$	−2.581

**Table 3:** Prediction results of machine_B.

Algorithm	MAE	MSE	MAPE	$R^{2}$
Tr-Predictor	$2.64\times 10^{-1}$	$9.05\times 10^{-2}$	$5.63\times 10^{-3}$	$9.95\times 10^{-1}$
ARIMA	$1.33\times 10^{-1}$	21.54	$7.91\times 10^{-2}$	−$7.73\times 10^{-2}$
CLA	$7.64\times 10^{-2}$	$8.60\times 10^{-1}$	$6.17\times 10^{-1}$	$1.07\times 10^{-2}$
AR	3.770	21.725	$1.64\times 10^{-1}$	$2.62\times 10^{-1}$
WANN	1.037	1.771	$1.13\times 10^{-2}$	$3.27\times 10^{-1}$
BA	2.253	16.532	$1.57\times 10^{-1}$	$9.86\times 10^{-1}$
C_L	$2.84\times 10^{-1}$	$1.20\times 10^{-1}$	$6.80\times 10^{-1}$	−2.04

**Table 4:** Prediction results of machine_C.

Algorithm	MAE	MSE	MAPE	$R^{2}$
Tr-Predictor	$2.22\times 10^{-1}$	$5.38\times 10^{-2}$	$7.11\times 10^{-3}$	$9.96\times 10^{-1}$
ARIMA	3.24	18.05372	$1.09\times 10^{-1}$	$5.02\times 10^{-2}$
CLA	$3.64\times 10^{-1}$	$4.99\times 10^{-1}$	1.875	−$1.99\times 10^{-2}$
AR	3.563	19.48	$1.18\times 10^{-1}$	−$1.86\times 10^{-2}$
WANN	1.43	3.44	$1.69\times 10^{-2}$	$7.37\times 10^{-1}$
BA	$5.53\times 10^{-1}$	$5.70\times 10^{-1}$	$9.91\times 10^{-1}$	$9.99\times 10^{-1}$
C_L	$3.58\times 10^{-1}$	$1.63\times 10^{-1}$	$8.41\times 10^{-1}$	−$3.37\times 10^{-1}$

**Table 5:** Comparison of different tasks under the MAE evaluation index.

Job ID	Algorithm						
	Tr-Predictor	ARIMA	CLA	AdaBoost.R2	WANN	BHT_ARIMA	CNN_LSTM
Job A	$4.09\times 10^{-4}$	$5.95\times 10^{-2}$	$1.006\times 10^{-1}$	$1.72\times 10^{-3}$	$5.33\times 10^{-4}$	$6.89\times 10^{-4}$	$2.09\times 10^{-1}$
Job B	$3.33\times 10^{-4}$	$1.33\times 10^{-1}$	$8.30\times 10^{-2}$	$8.36\times 10^{-3}$	$9.02\times 10^{-2}$	$2.89\times 10^{-3}$	$3.42\times 10^{-1}$
Job C	$1.07\times 10^{-3}$	$7.86\times 10^{-2}$	$5.52\times 10^{-2}$	$1.22\times 10^{-2}$	$2.05\times 10^{-1}$	$3.37\times 10^{-2}$	$2.44\times 10^{-1}$
Job D	$2.81\times 10^{-4}$	$1.12\times 10^{-2}$	$2.64\times 10^{-2}$	$1.04\times 10^{-3}$	$1.57\times 10^{-3}$	$3.72\times 10^{-4}$	$1.09\times 10^{-1}$
Job E	$2.08\times 10^{-4}$	$1.11\times 10^{-1}$	$6.43\times 10^{-2}$	$3.88\times 10^{-3}$	$3.25\times 10^{-3}$	$3.04\times 10^{-3}$	$5.16\times 10^{-1}$
Job F	$3.42\times 10^{-4}$	$7.24\times 10^{-2}$	$1.55\times 10^{-1}$	$5.06\times 10^{-3}$	$1.23\times 10^{-3}$	$3.01\times 10^{-3}$	$4.33\times 10^{-1}$
Job G	$1.70\times 10^{-3}$	$1.50\times 10^{-1}$	$1.64\times 10^{-1}$	$3.30\times 10^{-2}$	$2.14\times 10^{-2}$	$5.84\times 10^{-2}$	$5.42\times 10^{-1}$
Job H	$1.04\times 10^{-4}$	$7.57\times 10^{-2}$	$3.95\times 10^{-1}$	$3.11\times 10^{-4}$	$3.30\times 10^{-4}$	$1.58\times 10^{-4}$	$9.23\times 10^{-1}$
Job I	$4.61\times 10^{-4}$	$1.37\times 10^{-1}$	$2.48\times 10^{-3}$	$2.27\times 10^{-3}$	$8.32\times 10^{-4}$	$2.97\times 10^{-3}$	$1.86\times 10^{-1}$

**Table 6:** Comparison of different tasks under the MSE evaluation index.

Job ID	Algorithm						
	Tr-Predictor	ARIMA	CLA	AdaBoost.R2	WANN	BHT_ARIMA	CNN_LSTM
Job A	$1.70\times 10^{-7}$	$5.98\times 10^{-3}$	$1.25\times 10^{-2}$	$5.04\times 10^{-6}$	$4.80\times 10^{-7}$	$7.20\times 10^{-7}$	$5.52\times 10^{-2}$
Job B	$9.10\times 10^{-7}$	$5.70\times 10^{-2}$	$9.48\times 10^{-3}$	$1.36\times 10^{-4}$	$8.54\times 10^{-3}$	$1.41\times 10^{-5}$	$1.19\times 10^{-1}$
Job C	$1.77\times 10^{-6}$	$1.17\times 10^{-2}$	$5.10\times 10^{-3}$	$2.33\times 10^{-4}$	$2.86\times 10^{-3}$	$1.81\times 10^{-3}$	$6.55\times 10^{-2}$
Job D	$9.00\times 10^{-8}$	$2.15\times 10^{-4}$	$9.92\times 10^{-4}$	$1.31\times 10^{-6}$	$3.98\times 10^{-6}$	$2.70\times 10^{-7}$	$1.27\times 10^{-2}$
Job E	$6.00\times 10^{-8}$	$2.12\times 10^{-2}$	$5.95\times 10^{-3}$	$1.90\times 10^{-5}$	$1.62\times 10^{-5}$	$1.36\times 10^{-5}$	$2.76\times 10^{-1}$
Job F	$1.50\times 10^{-7}$	$8.06\times 10^{-3}$	$3.26\times 10^{-2}$	$6.02\times 10^{-5}$	$4.78\times 10^{-6}$	$1.83\times 10^{-5}$	$3.54\times 10^{-1}$
Job G	$4.71\times 10^{-6}$	$3.01\times 10^{-2}$	$4.08\times 10^{-2}$	$1.58\times 10^{-3}$	$7.07\times 10^{-4}$	$3.87\times 10^{-3}$	$3.17\times 10^{-1}$
Job H	$2.00\times 10^{-8}$	$9.32\times 10^{-3}$	$1.68\times 10^{-1}$	$1.60\times 10^{-7}$	$1.80\times 10^{-7}$	$7.00\times 10^{-8}$	$8.61\times 10^{-1}$
Job I	$3.10\times 10^{-7}$	$3.42\times 10^{-2}$	$8.88\times 10^{-2}$	$7.64\times 10^{-6}$	$9.90\times 10^{-7}$	$1.32\times 10^{-5}$	$3.74\times 10^{-2}$

**Table 7:** Comparison of different tasks under the MAPE evaluation index.

Job ID	Algorithm						
	Tr-Predictor	ARIMA	CLA	AdaBoost.R2	WANN	BHT_ARIMA	CNN_LSTM
Job A	$1.30\times 10^{-1}$	$2.36\times 10^{-1}$	$6.08\times 10^{-1}$	$7.88\times 10^{-1}$	$1.63\times 10^{-1}$	$1.71\times 10^{-1}$	$9.02\times 10^{-1}$
Job B	$6.19\times 10^{-3}$	$5.08\times 10^{-1}$	$2.21\times 10^{-1}$	$1.83\times 10^{-1}$	2.06	$6.29\times 10^{-2}$	$8.47\times 10^{-1}$
Job C	$9.78\times 10^{-3}$	$6.87\times 10^{-1}$	$4.24\times 10^{-1}$	$7.38\times 10^{-2}$	$4.45\times 10^{-1}$	$6.07\times 10^{-1}$	$7.02\times 10^{-1}$
Job D	$8.81\times 10^{-3}$	$8.95\times 10^{-2}$	$1.47\times 10^{-1}$	$3.26\times 10^{-2}$	$4.65\times 10^{-2}$	$1.03\times 10^{-2}$	$7.51\times 10^{-1}$
Job E	$4.13\times 10^{-3}$	$2.78\times 10^{-1}$	$1.43\times 10^{-1}$	$7.80\times 10^{-2}$	$7.07\times 10^{-2}$	$6.88\times 10^{-2}$	$9.18\times 10^{-1}$
Job F	$1.87\times 10^{-2}$	$6.17\times 10^{-1}$	$9.46\times 10^{-1}$	$9.10\times 10^{-1}$	$4.75\times 10^{-2}$	$1.66\times 10^{-1}$	$7.0\times 10^{-1}$
Job G	$1.51\times 10^{-2}$	$2.44\times 10^{-1}$	$9.99\times 10^{-1}$	$2.72\times 10^{-1}$	6.79	$4.63\times 10^{-1}$	$7.71\times 10^{-1}$
Job H	$1.41\times 10^{-2}$	$9.14\times 10^{-2}$	$4.78\times 10^{-1}$	$4.37\times 10^{-2}$	$4.54\times 10^{-2}$	$2.08\times 10^{-2}$	$7.86\times 10^{-1}$
Job I	$2.47\times 10^{-2}$	1.31	2.80	$1.32\times 10^{-1}$	$3.50\times 10^{-2}$	$1.32\times 10^{-1}$	$8.41\times 10^{-1}$

**Table 8:** Ablation experiment results of Job A.

Algorithm	MAE	MSE	MAPE	$R^{2}$
w/o LSTM	$1.96\times 10^{-3}$	$1.89\times 10^{-6}$	$3.23\times 10^{-1}$	$8.99\times 10^{-1}$
w/o Tr	$1.41\times 10^{-3}$	$2.64\times 10^{-6}$	$5.75\times 10^{-1}$	−$2.71\times 10^{-1}$
w/o TB	$7.56\times 10^{-3}$	$6.20\times 10^{-7}$	$1.81\times 10^{-1}$	$8.21\times 10^{-1}$
Tr-Predictor	$4.90\times 10^{-4}$	$1.70\times 10^{-7}$	$1.30\times 10^{-1}$	$9.44\times 10^{-1}$

**Table 9:** Ablation experiment results of Job G.

Algorithm	MAE	MSE	MAPE	$R^{2}$
w/o LSTM	$4.51\times 10^{-3}$	$6.10\times 10^{-6}$	$3.01\times 10^{-2}$	$9.95\times 10^{-1}$
w/o Tr	$2.43\times 10^{-2}$	$1.13\times 10^{-3}$	$2.01\times 10^{-1}$	$3.30\times 10^{-1}$
w/o TB	$3.26\times 10^{-2}$	$1.52\times 10^{-3}$	$1.78\times 10^{-1}$	−$7.06\times 10^{-1}$
Tr-Predictor	$1.70\times 10^{-3}$	$4.71\times 10^{-6}$	$1.51\times 10^{-2}$	$9.99\times 10^{-1}$

**Table 10:** Ablation experiment results of Machine_A.

Algorithm	MAE	MSE	MAPE	$R^{2}$
w/o LSTM	$2.01\times 10^{-1}$	$1.51\times 10^{-1}$	$6.85\times 10^{-3}$	$9.17\times 10^{-1}$
w/o Tr	5.79	58.11	$2.56\times 10^{-1}$	−$9.73\times 10^{-1}$
w/o TB	$7.56\times 10^{-1}$	$6.20\times 10^{-1}$	$1.81\times 10^{-1}$	$8.21\times 10^{-1}$
Tr-Predictor	$1.42\times 10^{-1}$	$3.01\times 10^{-2}$	$9.01\times 10^{-3}$	$9.996\times 10^{-1}$

## Data Availability

Google dataset: https://github.com/google/cluster-data, accessed on 11 April 2022; Alibaba dataset: https://github.com/alibaba/clusterdata/tree/v2018, accessed on 9 October 2022.
